# Network Structure of depressive symptomatology in elderly with Cognitive Impairment

**DOI:** 10.1002/alz.086353

**Published:** 2025-01-03

**Authors:** Jee Hyung Pyo

**Affiliations:** ^1^ Samsung medical center, seoul Korea, Republic of (South)

## Abstract

**Background:**

Cognitive impairment and loss of concentration often resides in both depressive disorder and cognitive dysfunction such as Alzheimer’s dementia and mild cognitive impairment (MCI). Also, substantial population of cognitive dysfunction experience depressive symptoms. This study finds out network structure of items of geriatric depression scale (GDS) in patients with cognitive dysfunction.

**Method:**

The sample of 5354 patients with cognitive dysfunction including both Alzheimer’s dementia [n = 1889], and MCI [n = 3464], were measured with GDS, geriatric depression scale, the extent of depressive symptoms. The scale is self‐administered questionnaire, which measures depression. Using exploratory graph analysis, network structure was analyzed as nodes and edges and centrality was represented as strength, betweenness, and closeness. The analysis was conducted between the subgroups of AD and MCI and evaluated by network comparison tests.

**Result:**

The centrality indices found that the most central item of GDS was the item 12 “worthlessness” in patients with cognitive impairment. Subgroup analysis showed the worthlessness was most central in GDS among AD patients while emptiness was the most central in MCI patients.

**Conclusion:**

The preventive approach and early intervention for worthlessness might be a crucial point in depressive symptoms in patients with cognitive dysfunction.

